# Are people with obsessive-compulsive disorder under-confident in their memory and perception? A review and meta-analysis

**DOI:** 10.1017/S0033291722001908

**Published:** 2022-10

**Authors:** Reuven Dar, Noam Sarna, Gal Yardeni, Amit Lazarov

**Affiliations:** School of Psychological Sciences, Tel Aviv University, Tel Aviv 69978, Israel

**Keywords:** Confidence, doubt, memory, metacognition, obsessive-compulsive disorder, perception

## Abstract

People with obsessive-compulsive disorder (OCD) tend to distrust their memory, perception, and other cognitive functions, and many OCD symptoms can be traced to diminished confidence in one's cognitive processes. For example, poor confidence in recall accuracy can cause doubt about one's memory and motivate repeated checking. At the same time, people with OCD also display performance deficits in a variety of cognitive tasks, so their reduced confidence must be evaluated in relation to their actual performance. To that end, we conducted an exhaustive review and meta-analysis of studies in which OCD participants and non-clinical control participants performed cognitive tasks and reported their confidence in their performance. Our search resulted in 19 studies that met criteria for inclusion in the quantitative analysis, with all studies addressing either memory or perception. We found that both performance and reported confidence were lower in OCD than in control participants. Importantly, however, confidence was more impaired than performance in participants with OCD. These findings suggest that people with OCD are less confident in their memory and perception than they should be, indicating a genuine *under-confidence* in this population. We discuss potential mechanisms that might account for this finding and suggest avenues for further research into under-confidence and related meta-cognitive characteristics of OCD.

## Introduction

Obsessive-compulsive disorder (OCD) was described more than a century ago as a disorder of doubt (Janet, 1903), with later theories of OCD agreeing on the central place of doubt in the disorder (e.g. Boyer & Lienard, 2006; Rapoport, 1989; Reed, 1985; Shapiro, 1965). In particular, people with OCD often distrust their memory, perception, and other cognitive functions, and many OCD symptoms can be understood in terms of diminished cognitive confidence. For example, individuals with OCD may attempt to reconstruct what they had experienced while driving to work, in an attempt to convince themselves that they have not accidentally run over an innocent pedestrian. Others may engage in repeated checking of doors and windows before leaving the house, because they cannot feel certain that they have properly closed them just a few minutes earlier.^†^^1^

Consistent with these observations, questionnaire-based studies have consistently found that people with OCD report a distrust in their own cognitive processes, particularly in their memory and perception (e.g. Cougle, Salkovskis, & Wahl, 2007; Hermans et al., 2008; Hermans, Martens, De Cort, Pieters, & Eelen, 2003; Nedeljkovic & Kyrios, 2007; Nedeljkovic, Moulding, Kyrios, & Doron, 2009). While these studies documented the general tendency of individuals with OCD to distrust their cognitive processes, several experimental studies directly examined the extent to which people with OCD feel confident about their performance in a variety of cognitive tasks. In these studies, participants with OCD and non-clinical control participants performed tasks that assess memory, perception, decision making, or general knowledge, and were then asked to rate their confidence in their own performance. Whereas some studies have found that participants with OCD reported lower levels of confidence as compared with control participants (e.g. Cougle, Salkovskis, & Thorpe, 2008; Dar, 2004; Dar, Rish, Hermesh, Taub, & Fux, 2000; Marton et al., 2019; Zitterl et al., 2001), no group differences were found in other studies (e.g. Cabrera, McNally, & Savage, 2001; Göz, Karahan, & Tekcan, 2016; Tekcan, Topçuoğlu, & Kaya, 2007).

Notably, most of the studies that assessed participants' subjective confidence in their performance have not examined these confidence ratings in relation to participants' actual performance. We are aware of only two studies that directly examined confidence ratings in relation to actual performance (Dar, 2004; Dar et al., 2000). In these studies, participants with OCD and non-clinical control participants completed a two-choice general knowledge test (e.g. ‘Which scientist is associated with quantum mechanics? 1. Albert Einstein; 2. Niels Bohr’). After indicating their answer to each item, participants were asked to rate the probability that their answer was correct. In addition, at the end of the test, participants were asked to estimate the number of items they had answered correctly. As predicted, participants with OCD were significantly less confident in their performance than non-clinical participants, both in terms of the mean probability that their answers were correct and in terms of their global estimation of their performance. In addition, direct comparisons of confidence and performance showed that participants with OCD underestimated their actual performance on the general knowledge test, which was in fact equal to that of the non-clinical participants.

As these results demonstrate, it is critical to assess not only subjective confidence, but also actual performance in these types of tasks. If performance is not assessed or considered (as was the case in a recent review; Ouellet-Courtois, Wilson, and O'Connor, 2018), it is impossible to rule out the possibility that the reduced confidence in OCD may actually reflect an accurate assessment of impaired performance. Put differently, the question is whether the lower reported confidence of people with OCD is in fact *too low* in relation to their actual performance in the relevant tasks. As documented above for confidence/doubt, however, the evidence regarding actual task performance of people with OCD in various cognitive domains is also mixed. Some of these studies have found the performance of OCD participants to be unimpaired (e.g. Boschen & Vuksanovic, 2007; Göz et al., 2016; Moritz et al., 2007; Tekcan et al., 2007), whereas others found OCD participants to display deficient performance in cognitive and perceptual tasks compared to controls (e.g. Moritz, Rietschel, Jelinek, & Bauml, 2011; Radomsky, Dugas, Alcolado, & Lavoie, 2014; Zitterl et al., 2001). Consistent with these latter reports, meta-analyses of actual cognitive performance in OCD concluded that people with OCD exhibit deficits in both verbal and non-verbal memory (Abramovitch, Abramowitz, & Mittelman, 2013; Shin, Lee, Kim, & Kwon, 2014).

The present review and meta-analysis were designed to evaluate the self-reported confidence of people with OCD in their cognitive performance in relation to their actual performance. Toward this aim, we reviewed all research articles that assessed both performance and confidence in participants with OCD as compared to non-clinical control participants. This allowed us to evaluate three major questions: (1) Is OCD associated with impaired performance, relative to controls, in tasks that assess memory and perception? (2) Do OCD participants report lower confidence in their performance in such tasks relative to control participants? and (3) Are individuals with OCD characterized with *under-confidence* in these domains? In other words, is their deficit in reported confidence larger than their deficit in actual performance?

## Method

### Search strategy

The systematic review protocol was registered in Prospero before undertaking the review (Dar, Lazarov, & Yardeni, 2020). The present report conforms to PRISMA guidelines (Moher, Liberati, Tetzlaff, & Altman, 2009). Studies were selected following a systematic search for publications at the end of July 2020, complemented by a second search in October of 2021 to check for any studies that might have been published in the interim period (none were found). The search covered PubMed, PsycNet, and ISI Web of Science. All relevant subject headings and free-text terms were used to represent OCD and confidence, using the following search terms (asterisk denotes truncation designed to capture grammatical variability): ‘obsessiv*, compulsiv*’ with ‘confiden*’, ‘certain*’, ‘doubt*’, ‘decision*’, ‘source monitoring’, ‘monitor*’, ‘meta cognit*’, ‘memory*’, ‘signal detect*’* and ‘calibrat*’. Additional records were identified by employing the Similar Articles feature in PubMed, and the Cited Reference Search in ISI Web of Science. Reference sections of review articles, book chapters, and studies selected for inclusion were searched for further studies.

### Search selection process

**Fig. 1.**
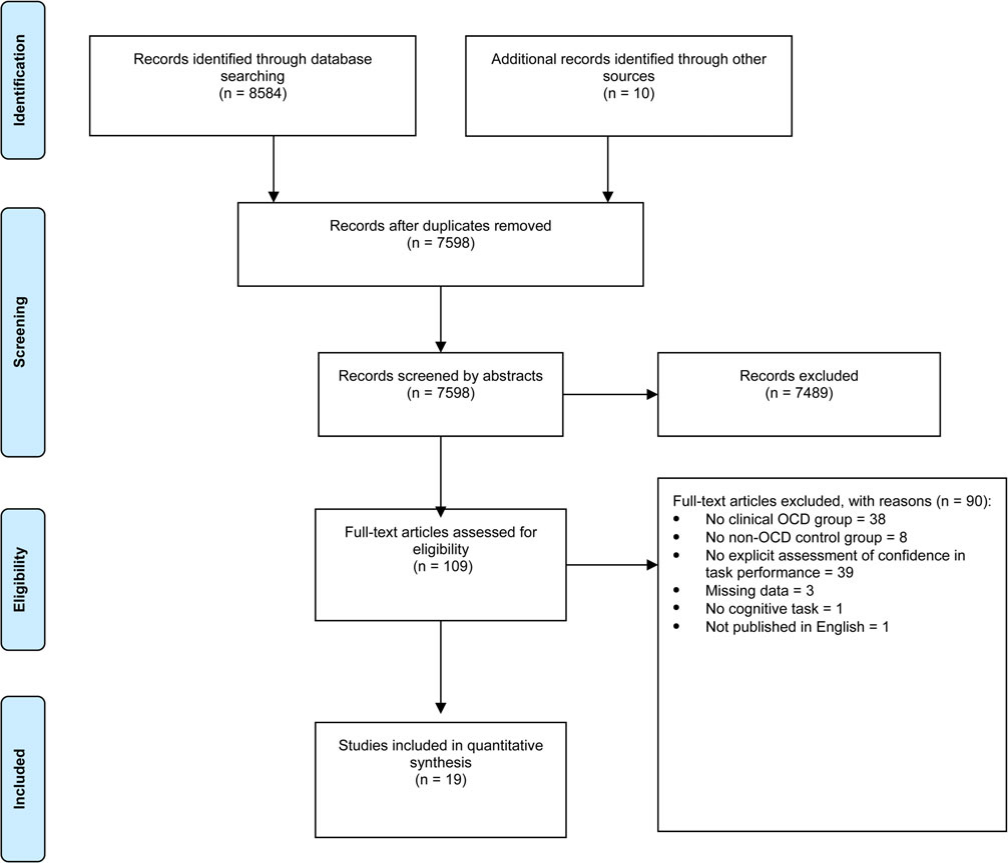
PRISMA flowchart of paper selection. Study selection process and reasons for exclusions.

Titles and abstracts were independently screened by two reviewers using Covidence systematic review software (Babineau, 2014), based on the inclusion and exclusion criteria outlined below. Discrepancies were resolved by discussion between the two reviewers. Full articles were then independently screened by each of the two reviewers. Inter-rater reliability was calculated, and where disagreements occurred, a consensus meeting was held to decide on study inclusion. Study selection process and reasons for exclusions are described in Fig. 1.

A study was included if: (1) OCD was assessed using a valid and accepted tool, including a diagnosis made by a clinician; (2) the performance of the OCD participants was compared to that of a control group – either healthy participants with no psychiatric disorder, or participants with another psychiatric disorder (e.g. anxiety disorders); (3) the study assessed both objective task performance and participants' explicit self-reported confidence as to their performance on the task; (4) the study included adult participants (aged >18); and (5) the study was published in English. Studies were excluded on the following grounds: (1) they were a review article, case study, or book chapter; (2) clinically relevant symptoms of OCD were not used in defining study groups; (3) the OCD group was not specifically identified; (4) lack of a non-OCD control group; (5) using ‘analog’ participants (i.e. non-selected participants, participants with subclinical OCD, or comparing high *v.* low scorers on OCD symptoms); and (6) assessing confidence only indirectly, with no explicit reporting of confidence levels.

### Data extraction

Data extraction was undertaken by the two reviewers and checked by the principal investigator (RD) for errors. Study characteristics extracted from reviewed studies included: (1) publication year; (2) sample size; (3) age of the OCD group; (4) gender ratio; (5) OCD subtype, if relevant; (6) comparison group/s (healthy controls, anxiety disorders)^2^; and (7) paradigm/task used (e.g. memory test, decision making, perceptual search, etc.). Table 1 lists the basic characteristics of the studies included in our analysis, and Table 2 details the performance and confidence measures used in each study.

**Table 1.** Study characteristics

Study	*N* OCD	*N* Control	% Females	Mean age (sd)	Task
Boschen and Vuksanovic (2007)	14	40	86	38.8 (−)	Stovetop repeated checking (free recall)
Cabrera et al. (2001)	21	21	52	32.2 (10.3)	Semantic integration
Cougle et al. (2008)	21	24	62	37.29 (10.0)	Reality monitoring
Dar et al. (2000)	20	20	30	36.85 (12.17)	General knowledge
Dar (2004)	15	15	13	–	General knowledge
Foa et al. (1997)	15	15	–	34.9 (12.9)	Noise rating task (with recognition phase)
Göz et al. (2016)	Checkers: 28	30	63–75	31.53 (10.81)	False memory task (DRM)
	Non-checkers: 32			34.34 (9.69)	
Karadag et al. (2005)	32	31	75	34.28 (10.95)	Recognition of sentences task (neutral and OC related)
Korotitsch (2004)	25	25	64	37.0 (12.9)	Memory recognition test – words were either read or heard
Marton et al. (2019)	26	44	–	–	The random dot motion task
McNally and Kohlbeck (1993)	Checkers: 12	12	58	31.4 (7.8)	Recognition of words and drawings
	Non-checkers: 12	12		31.2 (10.7)	
Moritz et al. (2006)	27	51	63	32.43 (8.86)	Source memory recognition task
Moritz et al. (2007)	28	28	54	33.07 (11.94)	Recognition of words
Moritz et al. (2009a)	43	46	74	32.74 (9.96)	Picture word memory test (recognition)
Moritz et al. (2009b)	32	32	72	34.00 (10.88)	Action memory task
Moritz et al. (2011)	30	20	60	30.03 (7.04)	Directed forgetting paradigm
Radomsky et al. (2014)	30	30	47	33.1 (10.2)	Stovetop repeated checking (free recall)
Tekcan et al. (2007)	Checkers: 25	27	–	30.76 (9.75)	General knowledge
	Non-checkers: 16			34.00 (11.30)	
Zitterl et al. (2001)	27	27	48	38.9 (12.3)	LGT-3: verbal, nonverbal and general memory

*Note*. The minus sign (−) represents data that were not available in the paper.

**Table 2.** Measures of accuracy and confidence in the studies included in the analysis

Study	Task	Measure of accuracy	Measure of confidence	Confidence scale
Boschen and Vuksanovic (2007)	Stovetop repeated checking (free recall)	Number of recall errors	Confidence in memory accuracy	Visual analog scale (1–10)
Cabrera et al. (2001)	Semantic integration	Sentence recognition – old/new	Confidence in recognition decision	For sentences judged old: +1 to +6 For sentences judged new: −1 to −6
Cougle et al. (2008)	Reality monitoring	Memory for whether actions were performed or imagined	Confidence in memory rating	Visual analog scale (0–100)
Dar et al. (2000)	General knowledge	Mean number of correct answers	Confidence in answer	5–100% with 5% intervals
Dar (2004)	General knowledge	Mean number of correct answers	Confidence in answer	5–100% with 5% intervals
Foa et al. (1997)	Noise rating task (with recognition phase)	Sentence recognition – old/new	Confidence in recognition	3-point scale with 1 indicating confidence and 3 indicating guessing
Göz et al. (2016)	False memory task (DRM)	Words recognition – old/new	Confidence in recognition	4-point scale: 1 – not confident at all to 4 – very confident
Karadag et al. (2005)	Recognition of sentences task (neutral and OC related)	Sentence recognition – old/new	Confidence in recognition	2-point scale: 1 indicating ‘I am completely confident’ and 0 indicating ‘I am not confident or I suspect’
Korotitsch (2004)	Memory recognition test – words were either read or heard	Words recognition – old/new	Confidence in recognition	3-point scale: 1 = not sure, 2 = fairly sure, 3 = sure
Marton et al. (2019)	The random dot motion task	Determine whether the dot cloud appeared to be moving to the right or left	Confidence in decision	7-point scale from 1 – low certainty to 7 – high certainty
McNally and Kohlbeck (1993)	Recognition of words and drawings	Recognition – old/new	Confidence in recognition	3-point scale: from 1 – guessing to 3 – certain
Moritz et al. (2006)	Source memory recognition task	Recognition – old/new/self-generated	Confidence in recognition	4-point scale: from 1 – guessing to 4 – entirely certain
Moritz et al. (2007)	Recognition of words	Words recognition – old/new	Confidence in recognition	6-point scale: entirely sure yes (100%), quite sure yes (80%), unsure yes (60%), unsure no (40%), quite sure no (20%), and entirely sure no (0%)
Moritz et al. (2009a)	Picture word memory test (recognition)	Recognition – old /new	Confidence in recognition	4-point scale: 1 = guessing, 2 = rather unsure, 3 = rather sure, 4 = entirely sure
Moritz et al. (2009b)	Action memory task	Recognition – verbal/nonverbal/novel (external source memory)	Confidence in recognition	4-point scale: from 1 – 100% certain to 4 – extremely uncertain
Moritz et al. (2011)	Directed forgetting paradigm	Recognition – old /new	Confidence was assessed together with the accuracy	6-point scale: 1 = 100% old, 2 = rather sure old, 3 = unsure old, 4 = unsure new, 5 = rather sure new, 6 = 100% new
Radomsky et al. (2014)	Stovetop repeated checking (free recall)	Recall – the number of knobs (out of 3) correctly recalled	Verbally rate confidence in decision	100-point scales, with 0 representing ‘not at all’ and 100 representing ‘extremely’
Tekcan et al. (2007)	General knowledge	Recognition – multiple choice	Confidence in recognition decision	0–100% with 20% intervals
Zitterl et al. (2001)	LGT-3: verbal, nonverbal and general memory	Scores on the LGT-3 test	Confidence in memory ability	5-point scale

*Note*: None of the studies provided feedback to participants on their performance in the tasks.

### Data analysis

We analyzed the data using Comprehensive Meta-Analysis, Version 3 (CMA; Borenstein, Hedges, Higgins, and Rothstein, 2015). Hedges' *g* was used as the effect size measure. The data on accuracy and on confidence were analyzed separately. Main effects (differences between OCD and control participants) were calculated using a random effect model, whereas the interaction effect (differences between the effects of accuracy and confidence) was calculated based on a mixed-effect model, as recommended by Borenstein and his colleagues (Borenstein, 2019; Borenstein, Hedges, Higgins, & Rothstein, 2009). In studies that measured accuracy and confidence using more than a single task (e.g. for both verbal and non-verbal stimuli, or for both OCD-relevant and irrelevant stimuli), the data of the two tasks were combined. In studies which included two groups of OCD participants (‘checkers’ and ‘non-checkers’), both of which were compared to the same control group, the *N* of the control group was divided by two to avoid inflation of type I error (Borenstein, 2019). In a series of secondary analyses, we examined the pattern of the results specifically in the subgroup of OCD ‘checkers’, as well as the potential effects of anxiety, depression, and medication status on the results.

Publication bias was examined (Sterne, Egger, & Smith, 2001), for both accuracy and confidence, using funnel plots with one-tailed Egger tests (Egger, Smith, Schneider, & Minder, 1997). The potential impact of such a bias on the results of the analysis was estimated using the Duval and Tweedie trim and fill method (Duval & Tweedie, 2000).

## Results

### Task performance and confidence

**Fig. 2.**
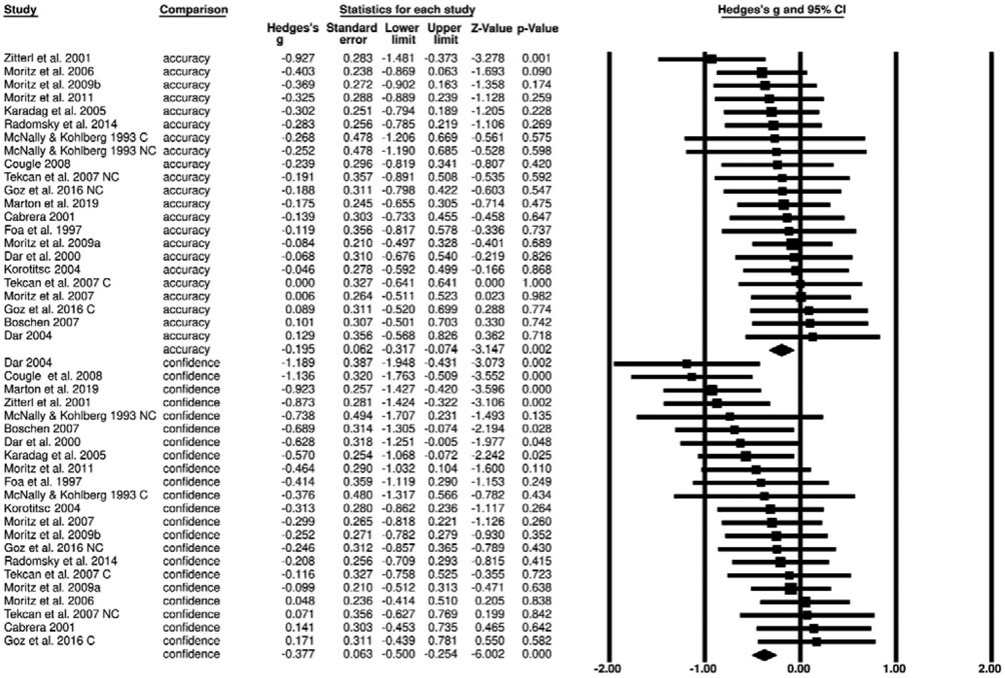
Effect sizes forest plot. Forest plot depicting effect sizes for accuracy and confidence. *Note.* Negative values of Hedges' *g* indicate lower scores of OCD participants as compared to control participants.

Our analysis indicated that overall, OCD participants performed significantly worse than non-clinical participants on the cognitive tasks examined in the included studies (*g* *=* −0.20, *Z* = −3.15, *p =* 0.002, 95% confidence interval (CI) [−0.32 to 0.01]). As can be seen in Fig. 2, this effect was relatively homogeneous across studies. This observation is supported by the non-significant *Q* statistic (12.786, df = 21, *p* = 0.916), indicating that the assumption of a common true effect in this body of studies could not be rejected.

Across studies, OCD participants were also significantly less confident than non-clinical control participants in their performance (*g* = −0.38, *Z* = −6.00, *p* < 0.001, 95% CI −0.50 to −0.25). In contrast to the performance results reported above, this effect was heterogeneous across studies, as indicated by the significant *Q* statistic (34.71, df = 21, *p* = 0.03).

As noted in the Introduction, the present meta-analysis also aimed at examining whether people with OCD are truly *under-confident*, that is, if they are less confident than they should be given their performance. In meta-analyses, the relevant statistic for answering this question is the *Z*-value that reflects the difference between the group effects of performance (*g* *=* −0.20) and confidence (*g* = −0.38). This value was statistically significant, *Z* = 1.99, *p* = 0.048, indicating that the overall difference between participants with OCD and non-clinical control participants in reported confidence was larger than the corresponding difference in performance.

We should note that the above value of *Z* for the interaction between performance and confidence is likely an underestimate of the true effect statistic. In CMA, this *Z* value assumes that the correlation between performance and confidence is zero (Borenstein et al., 2015). However, the few studies that reported the relevant correlation coefficients (Boschen & Vuksanovic, 2007; Cougle et al., 2008; Dar et al., 2000; Tekcan et al., 2007) found them to range between 0.16 and 0.91. As seen in Table 3, as the correlation coefficient between confidence and accuracy increases, the value of *Z* increases (and the *p* value decreases). If the correlation is assumed to be 0.3, *Z* becomes 2.38, with a *p* = 0.02. With a correlation of 0.5, which is in the middle of the range of correlations actually reported in this body of studies, the value of *Z* becomes 2.82, with *p* = 0.005.

**Table 3.** Significance tests of the differences between accuracy and confidence by assumed correlation between them

Assumed correlation	*Z*	*p* value
0	1.99	0.047
0.10	2.10	0.036
0.20	2.23	0.026
0.30	2.38	0.017
0.40	2.57	0.010
0.50	2.82	0.005
0.60	3.15	0.002
0.70	3.63	0>.001
0.80	4.45	0>.001
0.90	6.30	0>.001
0.99	1.42 × 10^8^	0>.001

### Secondary analyses

We conducted a secondary analysis including only samples of OCD participants that were selected on the basis of having primary checking symptoms (so-called ‘checkers; *N* = 8). The pattern of the results was very similar for this subgroup, but due to the small *N*, only the effect of confidence reached statistical significance (*g* = −0.55, *Z* = −3.44, *p* = 0.001, 95% CI −0.86 to −0.24).

Next, we examined whether confidence was related to anxiety or depression in our meta-analysis data. Unfortunately, very few studies reported the anxiety scores of the OCD participants. However, 14 studies did report depression scores for the OCD participants, either with Beck Depression Inventory (BDI) or the Hamilton Depression Rating Scale (seven studies each). To combine these two measures, we transformed all the scores into the BDI using the conversion table created by Furukawa et al. (2020). To test whether depression might have contributed to the differences between OCD and control participants in reported confidence, we computed the correlation between the effect sizes (i.e. Hedges’ *g* of the differences between OCD and control participants) and the unified depression scores. Depression was negatively correlated with the effect size for performance, *r* = 0.477, *p* = 0.021, but not for confidence, *r* = 0.148, *p* = 0.51, indicating that depression did not account for the differences in confidence between OCD and control participants in our data.

Finally, we attempted to assess the role of medication use in our results. Of the studies included in our analysis, only six reported the medication use status of the OCD participants (Dar et al., 2000; Korotitsch, 2004; Moritz, Jacobsen, Willenborg, Jelinek, & Fricke, 2006, 2007; Radomsky et al., 2014; Zitterl et al., 2001). Unfortunately, even these six studies did not provide sufficient details about the type and doses of the medications to allow an analysis of their potential effect on the dependent variables. It is worth noting, however, that two of the studies mentioned above (Moritz et al., 2006, 2007) did include medication status as a moderator in their analysis and found no effect of medication use on any of the dependent variables.

### Publication bias

With regard to confidence, an examination of the funnel plot suggested a small study effect, with smaller studies reporting larger effects. Consistent with this impression, the Begg and Mazumdar rank correlation test was significant, with a Kendall's tau correlation coefficient of −0.36 between sample size and effect size, *p*(1-tailed) = 0.008. Egger's regression was also significant in a one-tailed *t* test, *t*(21) = 2.39, *p* = 0.018. The trim and fill algorithm, which estimates publication bias, produced an adjusted effect size of −0.22, 95% CI −0.39 to −0.04. Notably, this adjusted CI did not include zero, so the differences between OCD and control participants in reported confidence remain significant after this correction. An examination of the funnel plot of the accuracy data did not suggest a small study effect, and none of the bias indicators were statistically significant.

## Discussion

The present meta-analysis is the first to examine the reported confidence of participants with OCD, as compared to non-clinical control participants, in relation to their performance on various cognitive tasks. We found that both performance and reported confidence were lower in OCD than in control participants. Importantly, however, our analysis indicates that confidence was more impaired than performance in participants with OCD. Put differently, OCD participants exhibit a larger reduction in confidence than in actual performance compared with non-clinical control participants. These findings imply that people with OCD are *under-confident* (i.e. less confident than they should be) regarding their performance.

The finding of cognitive under-confidence in OCD participants is particularly important in light of the observation that humans generally tend to be *over*-confident in their abilities and performance (e.g. Ehrlinger, Mitchum, & Dweck, 2016; Koriat, Lichtenstein, & Fischhoff, 1980; Moore & Healy, 2008; Prims & Moore, 2017). As a rule, then, the confidence of most people in their performance and abilities is too high given their actual performance or abilities. Overconfidence has been documented across many domains – people are likely to overestimate their success in a test, their driving skills, or their chances of doing well in the stock market. Experimental studies using methods such as those described in the Introduction, where participants answer multiple choice questions, consistently found participants from the general population to be overconfident in their answers. For example, in a series of studies by Fischhoff, Slovic, and Lichtenstein (1977), even answers that participants were absolutely certain about (i.e. rated the probability that they were correct as 1.00) were in reality erroneous about 20% of the time. Participants in these studies were clearly unaware that their confidence in their performance was excessive, as evidenced by their willingness to bet money on their answers. Overconfidence is assumed to be a universal bias, proposed to have an evolutionary basis and to be adaptive for optimal functioning (Johnson & Fowler, 2011; Tobena, Marks, & Dar, 1999). Against this backdrop, the current findings of under-confidence in individual with OCD appears to be unique,^3^ suggesting not merely a lack of a protective normative bias in OCD, but rather the existence of a negative bias in the opposite direction.

Asking someone to appraise their own performance on a task in the absence of feedback presents a challenging task for most people. Indeed, a recent meta-synthesis found that the mean correlation between self-evaluation and actual performance reported in meta-analyses across a variety of performance domains was only moderate (*r* = 0.29; Zell and Krizan, 2014). A recent novel model of OCD suggests that in OCD, these internal appraisals may be particularly challenging. According to the Seeking Proxies for Internal States (SPIS) model (Dar, Lazarov, & Liberman, 2021), OCD symptoms are associated with attenuated access to internal states. Evidence supporting this hypothesis was obtained in relation to several internal states, including muscle tension (Lazarov, Dar, Liberman, & Oded, 2012b; Lazarov, Liberman, Hermesh, & Dar, 2014), emotions (Dar, Lazarov, & Liberman, 2016; Lazarov, Friedman, Comay, Liberman, & Dar, 2020), interoception (Ezrati, Friedman, & Dar, 2019; Ezrati, Sherman, & Dar, 2018), and a sense of understanding (Dar, Eden, van Dongen, Hauschildt, & Liberman, 2019). As estimating one's performance requires accessing an internal state (in the current review, particularly one's memory), the under-confidence seen in our analysis might be an expression of a general difficulty in accessing these states in OCD. Moreover, according to the SPIS model, the process of self-doubt that results from attenuated access to internal states may lead, in turn, to actual performance deficits. For example, instructions that undermined unselected participants' confidence in their ability to assess their own muscle tension (Lazarov, Cohen, Liberman, & Dar, 2015; Lazarov, Dar, Liberman, & Oded, 2012a) or emotions (Dar et al., 2016) led to actual impairment in the relevant task performance. These findings may suggest that the performance deficits in cognitive tasks documented in our meta-analysis (see also Abramovitch et al., 2013; Shin et al., 2014) may be partially caused by the impaired confidence of OCD participants.

From another theoretical perspective, over- and under-confidence are both examples of biases in metacognition (i.e. the process of monitoring one's own cognitive processes). Resonating with our findings, several researchers have suggested that OCD is associated with deficits in metacognition (e.g. Ben Shachar, Lazarov, Goldsmith, Moran, & Dar, 2013; Hauser, Allen, Consortium, Rees, & Dolan, 2017). In current theorizing, metacognition is typically broken down into two distinct components – *bias*, as reported here, and *sensitivity*, which is the ability to discriminate accurate from inaccurate performance (Fleming & Lau, 2014). According to a recent hierarchical model of metacognition (Seow, Rouault, Gillan, & Fleming, 2021), metacognition is shaped through an interplay between multiple hierarchical levels of metacognitive estimation; hence a confidence bias, as detected in our analysis, can result from an impaired local level sensitivity component affecting up-stream a more global bias. As far as we know, only two experimental studies have attempted to measure specific components of metacognition in relation to OCD (Ben Shachar et al., 2013; Hauser et al., 2017). Unfortunately, these studies, which relied on non-clinical samples, used different tasks and assessment methods and reached contradicting results – whereas Hauser et al. (2017) concluded that high-OCD participants had deficient metacognitive sensitivity, Ben Shachar et al. (2013) did not.

The finding of under-confidence in OCD may also indicate that in judging their own behavior, participants with OCD were more influenced by their prior beliefs than by directly observed data (such as task difficulty and task performance). As noted above, several studies have documented that people with OCD tend to distrust their cognitive functions (e.g. Cougle et al., 2007; Hermans et al., 2008, 2003; Nedeljkovic et al., 2009; Nedeljkovic & Kyrios, 2007). Such prior beliefs can have a top-down effect on the interpretation of sensory evidence, diminishing its weight in the inferential process (Knill & Pouget, 2004; Sherman, Seth, Barrett, & Kanai, 2015). Similar processes have been integrated into a recent hierarchical model of metacognition (Seow et al., 2021), which assumes that global self-belief can influence downstream the local components of performance estimation, leading to biased meta-cognitive estimates.

At present, the ideas sketched above are clearly speculative, and their examination requires much further study. Specifically, research using newly developed computational models (Hauser et al., 2017; Seow & Gillan, 2020; Vaghi et al., 2017) might lead to more precise delineation of the underlying components and processes making up the apparent metacognitive deficit in OCD. We believe that a better mapping of these metacognitive deficits is important for understanding the pervasive doubt experienced by many individuals suffering from OCD. Down the road, it might also guide attempts to help OCD clients to cope with their doubts and related symptoms, such as repeated checking and requests for reassurance. For example, understanding the role of difficulties in accessing internal states on confidence in cognitive performance may lead to interventions designed to improve access to internal states. To the extent that people with OCD rely on prior beliefs regarding their cognitive function rather than on observed or experienced evidence, they might be encouraged and even trained to increase reliance on bottom-up processes by focusing on their present experience (e.g. by learning mindfulness techniques; see discussion in Dar et al., 2021).

In conclusion, the present review and meta-analysis indicates that people with OCD display both performance deficits and under-confidence in a variety of cognitive tasks. Future research might provide more fine-tuned data in regard to both of these effects, explore the causal relationships between them, and suggest ways in which such findings can be used to facilitate the understanding and treatment of people with OCD.

## Footnotes

† The notes appear after the main text.

## Notes

1 Notably, checking may be motivated by other factors, such as fear of causing harm, the wish to avoid guilt, or reducing ‘not just right’ feelings (for a recent review see Strauss et al., 2020).

2 In fact, only one study of those included in the analysis (Dar et al., 2000) incorporated a comparison group with anxiety disorders; all others included only non-clinical control groups.

3 While other mental disorders may also be associated with abnormalities in confidence, most of these abnormalities are in the direction of exaggerated overconfidence (Hoven et al., 2019). The only exception appears to be major depression, but extant evidence is sparse and inconsistent.
